# Hyponatremia: An Unusual Presentation in a Neonate With Chromosome 1q21.1 Deletion Syndrome

**DOI:** 10.3389/fped.2018.00273

**Published:** 2018-10-11

**Authors:** Bakri Alzarka, Rachel Usala, Matthew T. Whitehead, Sun-Young Ahn

**Affiliations:** ^1^Department of Nephrology, Children's National Health System, Washington, DC, United States; ^2^The George Washington University School of Medicine, Washington, DC, United States; ^3^Departments of Medicine and Pediatrics, MedStar Georgetown University Hospital, Washington, DC, United States; ^4^Department of Radiology, Children's National Health System, Washington, DC, United States

**Keywords:** hyponatremia, neonate, hypothalamus, tolvaptan, SIADH

## Abstract

Chromosome 1q21.1 deletion syndrome is associated with a wide variety of clinical features including mild to moderate mental retardation, microcephaly, cardiac abnormalities, and cataracts. We report an unusual case of a premature neonate with persistent hyponatremia, markedly elevated plasma arginine vasopressin level (32.7 pg/mL), and clinical findings consistent with the syndrome of inappropriate antidiuretic hormone secretion (SIADH). The patient, who also had microcephaly and dextrocardia, was subsequently diagnosed with chromosome 1q21.1 deletion syndrome. Further evaluation revealed hypothalamic abnormalities, features not previously described with this syndrome. To our knowledge, this is the first report of SIADH associated with congenital hypothalamic anomalies in a neonate with chromosome 1q21.1 deletion syndrome. We also report our experience using tolvaptan, a vasopressin receptor antagonist, in this patient to effectively maintain eunatremia.

## Introduction

Arginine-vasopressin (AVP) is a hormone that is produced by the magnocellular neurosecretory neurons in the hypothalamus and released from the posterior pituitary gland. AVP binding to AVP V2 receptors (AVPR2) leads to insertion of aquaporin 2 water channels on the apical surface of the principal cells of the cortical collecting duct, which increases water permeability and reduces renal water excretion (1).

AVP secretion can be physiologically stimulated by conditions such as hyperosmolality or hypotension, which signal the body's need for water conservation, or pathologically in the syndrome of inappropriate antidiuretic hormone secretion (SIADH). SIADH, as defined by the classic Bartter-Schwartz criteria, is hyponatremia associated with serum hypo-osmolality, less than maximally diluted urine, and no evidence of volume depletion (2). Several conditions can cause SIADH, including tumors, central nervous system (CNS) disorders, medications, pulmonary disease, and glucocorticoid deficiency (3).

Chromosome 1q21.1 deletion syndrome (OMIM#612474) is associated with neurodevelopmental disorders and a wide variety of clinical abnormalities, including cataracts and cardiac defects (4). However, chromosome 1q21.1 deletion syndrome has not previously been reported in association with congenital hypothalamic anomalies or SIADH to our knowledge. Moreover, congenital CNS anomalies causing neonatal SIADH is rare (5). The first-line treatment for SIADH is fluid restriction (6), which is challenging in infants whose nutrition consists predominantly of fluids. Other treatment options for SIADH include the use of furosemide and sodium supplementation. Tolvaptan, an oral AVPR2 antagonist, is also used to correct mild to moderate hyponatremia in patients with SIADH (7). Clinical trials of the safety and effectiveness of tolvaptan in children and adolescents is currently ongoing (8).

## Case presentation

A 6 week-old African-American female was born to nonconsanguineous parents. The G2P1 mother had a surgically removed pituitary prolactinoma prior to her pregnancy, which was complicated by gestational diabetes and hypertension.

The infant was born via spontaneous, vaginal delivery at 34 weeks and 2 days gestation. Birth weight was 2,091 grams (28%ile), length was 42.5 cm (12%ile), head circumference was 30 cm (30%ile), and Apgar scores were 6 and 8 at 1 and 5 min, respectively. Although intubated shortly after birth due to weak respiratory effort, she was extubated soon after without complications. Her physical exam was significant for heart sounds on the right side of her chest, and an echocardiogram demonstrated dextrocardia and a small atrial septal defect. The rest of her physical exam showed a well-appearing newborn, with normal vital signs, moist mucous membranes, appropriate capillary refill time, and normal infantile genitalia. Ultrasonography showed situs inversus and a duplicated right renal collecting system. Microarray results were consistent with chromosome 1q21.1 deletion syndrome.

### Investigations

Hyponatremia (serum sodium 128 mmol/L) was identified shortly after birth, although other electrolytes and kidney function were normal. Serum osmolality was low at 270 mOsm/kg (normal 275–295), urine osmolality was inappropriately elevated at 455 mOsm/kg, and urine sodium was also relatively high at 123 mEq/L. Plasma AVP level was significantly elevated at 32.7 pg/mL (normal 1–11). These findings were consistent with a diagnosis of SIADH.

Further evaluation showed a normal plasma aldosterone at 6 ng/dl (normal 1–197) and a normal 17-hydroxyprogesterone level on newborn screen. A random cortisol level was low at 3 mcg/dl (normal 5–25), but ACTH stimulation test was normal with a peak cortisol response of 48 mcg/dl. Thyroid function test showed a normal free T4 level of 1.5 ng/dl (normal 0.76–1.46) and a normal TSH level at 2.9 mIU/L (normal 0.7–11.0). Liver studies, triglycerides, and serum albumin levels were also normal.

**Figure 1:**
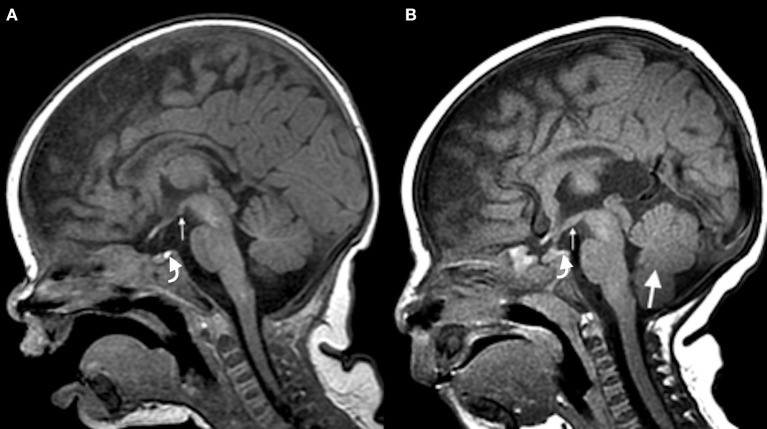
Sagittal T1-weighted brain MRI. **(A)** Normal MRI showing a homogeneous and marked signal hyperintensity pattern of the sellar region (curved arrow) in a 2 month-old infant and a normal hypothalamus (small arrow). **(B)** Patient's MRI showing a diminutive and barely discernible posterior pituitary hyperintensity (curved arrow), hypothalamic malformation (small arrow), and hypoplastic vermis (large arrow).

Brain magnetic resonance imaging (MRI) revealed a markedly diminutive posterior pituitary hyperintensity on T1-weighted images (Figure 1), a malformed sella turcica with otherwise normal adenohypophysis, posterior/inferior hypothalamic malformation with hypoplastic and incompletely separated mammillary bodies, mild vermian hypoplasia, mild cerebral white matter volume loss or hypoplasia, and mild microencephaly.

### Treatment and outcome

**Figure 2:**
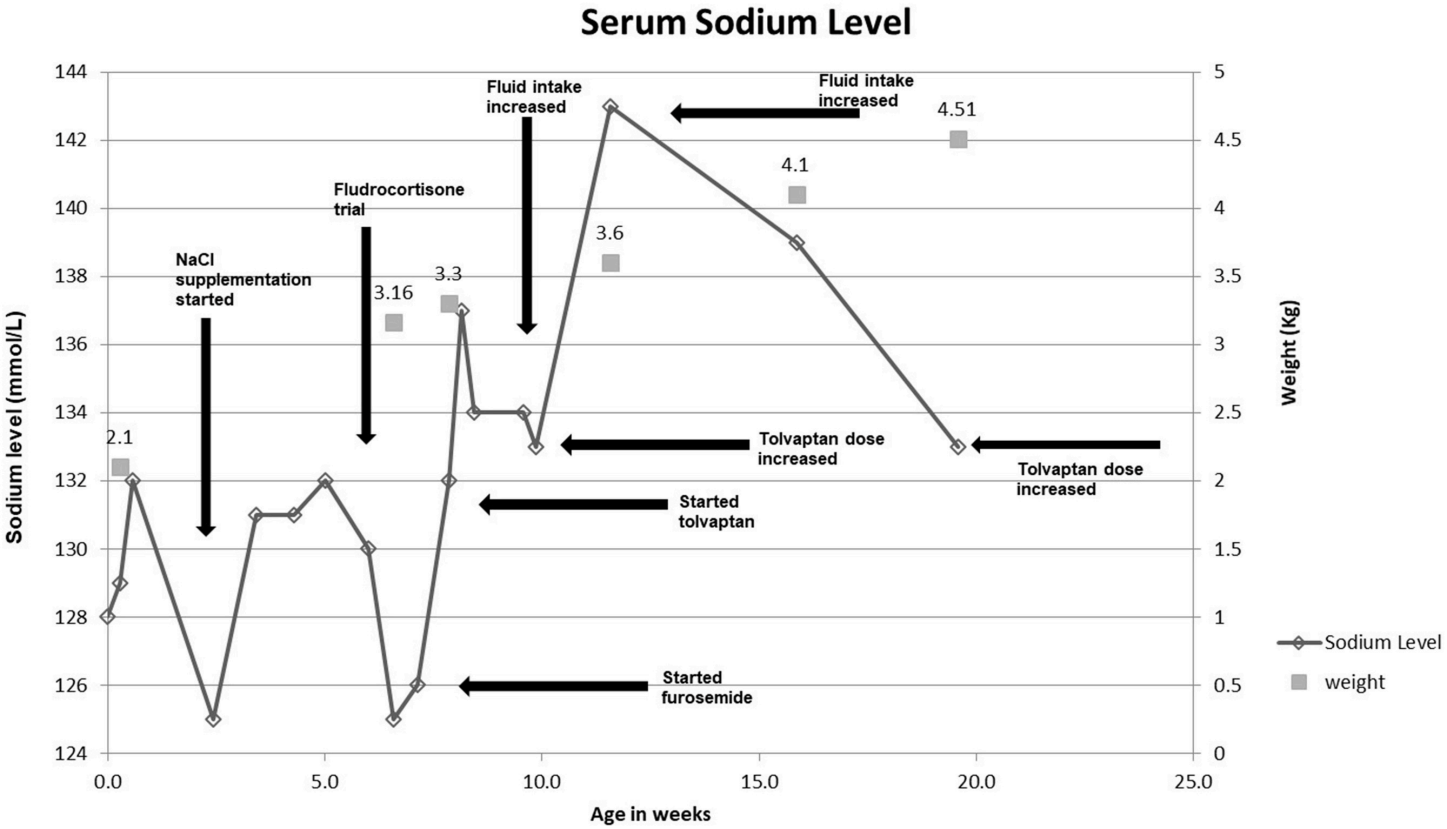
Serum sodium levels and weight of the patient as a function of time throughout the course of the infant's care.

Figure 2 demonstrates the serum sodium levels and weight of the infant as a function of time throughout the course of the infant's care. Sodium supplementation of up to 12 mEq/kg/day and fluid restriction resulted in minimal improvement in the serum sodium level, and actually led to mild hypertension. Hyponatremia not associated with hyperkalemia or metabolic acidosis renders isolated mineralocorticoid deficiency a less probable diagnosis; however, due to the persistent hyponatremia, a two-day trial of fludrocortisone was attempted but failed to improve the infant's serum sodium levels. Furosemide 2 mg/kg/day was added at 7 weeks of age with little effect. Tolvaptan (crushed and mixed with formula) was started at 0.05 mg/kg/day at 7 weeks of age, which resulted in normalization of her serum sodium levels. Sodium supplementation was subsequently weaned over the next few days, which resulted in blood pressure normalization. The daily fluid intake of the infant was successfully increased to provide the required caloric intake without development of hyponatremia.

The patient was discharged normotensive from our center at the age of 8 weeks with a normal serum sodium level of 136 mmol/L. Her discharge prescriptions included 4 mg of furosemide twice daily (2.4 mg/kg/day), 4 mEq of sodium chloride three times daily (3.75 mEq/kg/day), and 0.3 mg of tolvaptan once daily (0.09 mg/kg/day). She was discharged on a fluid restriction of 137 mL/kg/day of 28 kcal/oz of fortified formula to support her growth as a premature infant.

As the patient grew and gained weight, her daily formula intake was increased to maintain adequate caloric intake. Her tolvaptan dose has been incrementally increased with the increased fluid volume to prevent hyponatremia. Her weight and height have continued to track along the 1st percentile. Her head circumference is tracking below the 1st percentile.

## Discussion

Our patient had phenotypic features consistent with chromosome 1q21.1 deletion syndrome including microcephaly, cardiac abnormalities and other dysmorphic features (4). Chromosomal microarray showed an approximately 2.7 Mb interstitial deletion of 1q21.1-q21.2, confirming the diagnosis. Analysis of the deletion was performed using the UCSC Genome Browser, genome assembly version GRCh37/hg 19 (Feb. 2009). The deletion included segments of the genes *LOC100288142* and *NBPF10*, complete copies of *GPR89C, PDZK1P1, NBPF24, NBPF11, HYDIN2, NBPF12, LOC728989, NBPF13P, PRKAB2, PDIA3P, FMO5, CHD1L, LINC00624, BCL9, ACP6, GJA5, GJA8, GPR89B, NBPF8, MIR5087, FLJ39739, PPIAL4B, PPIAL4A, PPIAL4D, PPIAL4F, NBPF14*, and *NBPF9*, and a segment of *NBPF15*. Some mutations in these genes correspond to known phenotypic features of the chromosome 1q21.1 deletion syndrome. For example, mutations in *GJA5*, expressed in the heart, are linked to cardiac defects (9). Deletions in *GJA8*, which is expressed in the ocular lens, are linked to cataract formation (10). Furthermore, mutations in the neuroblastoma breakpoint family (NBPF) genes, expressed in the brain, are linked to 1q21-associated microcephaly (11). However, none of the genes included in the patient's 1q21.1 deletion are reported in association with hypothalamic or pituitary malformations. The reference sequence used in this analysis (UCSC Genome Browser assembly) contains multiple assembly gaps within sub-band 1q21.1 due to its complex genomic architecture. The incomplete assembly renders genotype-phenotype correlation challenging and thus limits our analysis, as these gaps may contain genes that could explain the features that we observed in our patient such as the hypothalamic malformation. Parental array studies were recommended to determine whether the deletion has a familial or de-novo origin; however, parents refused genetic studies as they are asymptomatic.

Since various conditions can cause SIADH in children (3), our patient required thorough evaluation before we determined that her congenital CNS abnormalities were the most likely etiology of her SIADH. SIADH has been associated with CNS disorders including brain trauma, meningitis, encephalitis, tumor, and hypoxia. However, reports of congenital CNS anomalies causing early SIADH are very rare; one such case describes a newborn with alobar holoprosencephaly who developed refractory SIADH (12). Another case report describes SIADH associated with a suprasellar arachnoid cyst (5). However, to our knowledge, congenital hypothalamic anomalies have not been described in association with SIADH.

Our patient's brain MRI showed multiple abnormalities including posterior/inferior hypothalamic malformation and a markedly diminutive posterior pituitary hyperintensity on T1-weighted images. Posterior pituitary hyperintensity on T1-weighted images is normally observed in the posterior aspect of the sella turcica. Studies suggest that this area of hyperintensity results from the storage of AVP within neurosecretory granules (13). We therefore speculate that the markedly diminutive posterior pituitary hyperintensity on T1-weighted images observed in our patient is related to AVP being abnormally released and not stored in the posterior pituitary gland. Indeed, absence of the high intensity signal in the posterior pituitary has been observed in patients with SIADH (14).

Also of interest in this case was the level of elevation in AVP observed. SIADH patients generally have plasma AVP levels that are in the normal range for normo-osmolality, but inappropriately elevated relative to hypo-osmolality. In one case series of 79 patients with SIADH, 80% of the study subjects had vasopressin levels that were inappropriately elevated relative to hypo-osmolality. Only 7 of the 79 patients in this series had AVP levels over 30 pg/mL (15). Many CNS disorders are associated with SIADH, but AVP levels are rarely reported above 10 pg/mL (16). Our patient lacked any known stimuli for AVP secretion, such as hypotension, hypovolemia, hypoglycemia, or increased plasma osmolality levels. Therefore, the presentation of our patient with congenital CNS anomalies and pathologically elevated AVP supports the hypothesis that AVP hypersecretion can occur by excitation of excitatory pathways via irritative foci, or disruption of opioid, gamma-amino butyric, or similar inhibitory pathways of AVP secretion that have yet to be elucidated (17–19).

An increasing number of studies report an association between asymptomatic chronic hyponatremia in children and serious complications such as sensorineural hearing loss (20) and defective growth in premature infants (21, 22). Premature infants are at greater risk for hyponatremia from high urinary excretion of sodium during the first 2–3 weeks of life (23). The elevated sodium losses may be due to an impaired ability of the immature kidney to regulate solute and water homeostasis as a result of low expression of Na+/K+-ATPase and an inadequate response to hormones responsible for regulating tubular sodium excretion (23, 24). These mechanisms may have partially contributed to the hyponatremia seen in our patient shortly after birth; however, our patient had persistent severe hyponatremia beyond 3 weeks of life that is typically not seen in infants of similar gestational age. Studies have shown that prevention of hyponatremia with early sodium supplementation resulted in improved growth in premature infants (21, 22). Therefore, although our patient was asymptomatic with chronic hyponatremia, treatment was strongly thought to be warranted.

Our patient responded incompletely to multiple modalities directed toward achieving normonatremia, including fluid restriction, sodium supplementation, and furosemide. Thus, we initiated an off-label pharmaceutical, tolvaptan, which resulted in normalization of our patient's serum sodium level. Tolvaptan is an oral AVPR2 antagonist that is used to correct hyponatremia in euvolemic or hypervolemic patients (25). It competitively inhibits binding of AVP to AVPR2 in the principal cells of the renal collecting ducts, thus preventing water reabsorption and inducing diuresis with proportionally more free water than sodium loss (7, 26). A few reports have addressed tolvaptan use in children, including a recent case series that demonstrated safe and effective use of tolvaptan over 3–4 years in two children and over 3 months in one child with chronic hyponatremia from SIADH (27). Another report described the use of tolvaptan in two infants with SIADH; tolvaptan was started at 2 and 4 months of age, respectively, with effective normalization of serum sodium values and no side effects (28). Similarly, we have been able to show the effective use of tolvaptan to treat SIADH in an infant.

Due to the limited information on tolvaptan use in children, little is known about its adverse effects in the pediatric population, especially in infants. However, the few pediatric reports available to date, including the case series described above, have reported minimal to no side effects for tolvaptan (27, 28). In a study of 34 pediatric patients (ages 2–202 months), who received tolvaptan for congestive heart failure and fluid overload, 6 patients experienced thirst and a dry mouth, and 1 patient had a mild increase in liver enzymes (29). In adults, potential adverse effects of tolvaptan include rapid overcorrection of serum sodium levels, liver injury, thirst, dry mouth, urinary frequency, and fatigue (25). Since there is limited information available regarding tolvaptan use in children and due to the concerns for liver toxicity with long-term use (25), close monitoring for side effects is warranted. Tolvaptan should be initiated in a hospital setting to monitor for hypovolemia, dehydration, and rapid overcorrection of serum sodium levels which can lead to neurological complications (25, 26).

In summary, we describe a rare case of congenital SIADH associated with hypothalamic anomalies in a neonate with chromosome 1q21.1 deletion syndrome. Our collective findings highlight the importance of considering SIADH as an etiology for hyponatremia in patients who have features consistent with chromosome 1q21.1 deletion syndrome. Although the causality cannot be proved, brain imaging should also be performed to screen for congenital hypothalamic malformations as a possible cause of SIADH in these patients. In addition, this report suggests that tolvaptan may be an effective therapeutic option for infants with SIADH.

## Ethics statement

Written informed consent was obtained from the parent of the patient for the publication of this case report.

## Author contributions

BA and RU contributed to the conception, data acquisition/analysis, and writing of the manuscript. MW provided the radiology findings and revised the manuscript. S-YA contributed to the conception, data analysis, writing and revision of the manuscript.

### Conflict of interest statement

The authors declare that the research was conducted in the absence of any commercial or financial relationships that could be construed as a potential conflict of interest.
